# Fatal aluminium phosphide poisoning

**DOI:** 10.1515/intox-2015-0010

**Published:** 2015-06

**Authors:** Mahesh Chand Meena, Sachin Mittal, Yashoda Rani

**Affiliations:** Forensic Medicine and Toxicology, Lady Hardinge Medical College, New Delhi, India

**Keywords:** aluminium phosphide, phosphine, poisoning, suicide

## Abstract

Aluminium phosphide (AlP) is a cheap solid fumigant and a highly toxic pesticide which is commonly used for grain preservation. AlP has currently aroused interest with a rising number of cases in the past four decades due to increased use for agricultural and non-agricultural purposes. Its easy availability in the markets has increased also its misuse for committing suicide. Phosphine inhibits cellular oxygen utilization and can induce lipid peroxidation. Poisoning with AlP has often occurred in attempts to commit suicide, and that more often in adults than in teenagers. This is a case of suicidal consumption of aluminium phosphide by a 32-year-old young medical anesthetist. Toxicological analyses detected aluminium phosphide. We believe that free access of celphos tablets in grain markets should be prohibited by law.

## Introduction

Aluminium phosphide (AlP) is a solid fumigant which has been extensively used since the 1940s. In India it is marketed as a tablet of celphos, alphos, quick phos, phostoxin, *etc.* (Chopra *et al*., 1986). In Iran it is known as the rice tablet (Mehrpour *et al*., 2010) that can be purchased in local shops.

This highly toxic chemical is cheap and usually formulated in tablets or pellets, granules and as powder. Upon contact with moisture, aluminium phosphide liberates phosphine, which is the active pesticidal component (Sudakin, 2005). Human toxicity, which is usually acute, occurs due to the toxic effects of phosphine released in the stomach after ingestion of aluminium phosphide. Phosphine is widely absorbed from the gastrointestinal tract. It emerges as a poison of suicidal deaths as this pesticide has no effective antidote, is cheap, freely available, and is a ‘sure agent of death’ (Nagar, 1985, Singh *et al*., 2003).

In India, this poisoning was not known before 1980. The first case in India was reported in 1981 from M.G.M. Medical College, Indore (Kabra *et al*., 1988). The incidence of the poisoning has been increasing steadily and is now the commonest mode of suicide in the agricultural community in northern India (Ranga et.al, 2004). Overall mortality in cases of aluminium phosphide poisoning varies between 70–100%. It is higher in those who consume more than two tablets and none of the patients who had ingested more than 3 tablets survived (Siwach *et al*., 1988, Singh *et al*., 1985, Raman *et al*. 1985). Suicide was the most common cause of death with 94%, followed by accident with 5% of cases, and homicide accounted for 1% of deaths (Gupta *et al*., 2006).

In France, aluminium phosphide is used for crop protection and because of its toxicity to humans and domestic animals, the use of AlP is strictly controlled and it cannot be freely supplied.

## Case report

This is a case of suicidal consumption of aluminium phosphide by a 32-year-old young medical anesthetist who was under training at a reputed hospital. He visited the walled city and hired a room in a suburb. He was determined to consume the lethal dose but due to the prolonged effect, he survived initially and started vomiting, alerted the hotel staff complaining of an upset stomach and was rushed to hospital. There on arrival he gave his details, fell unconscious and later succumbed to the effects of the poison, which was identified by the remaining unused tablets later recovered from his luggage at the hotel. The reason which was revealed by the investigating officer later was depression as he belonged to a disturbed family with problems that made him take this extreme step.

**Figure 1:**
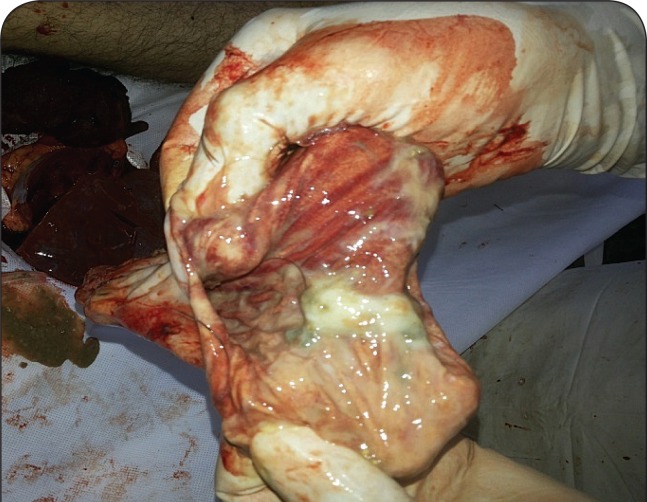
Mucosa of the stomach – congested and hemorrhagic.

On *post-mortem* examination, cyanosis was present over the nail bed. On opening the body, fishy or garlicky smell of phosphine gas was present. All the internal organs were congested with petechial hemorrhages. The lungs were congested and edematous with rupture of alveoli, gastrointestinal mucosal congestion (Figure 1), and petechial hemorrhages on the surface of the liver and brain. On toxicological analysis, aluminium phosphide was detected.

## Discussion

Aluminium phosphide is marketed in India as tablets of Celphos, Quickphos, *etc.* It is available in small and large packs containing grayish-white tablets weighing about 3 g each, containing 56% aluminium phosphide and 44% aluminium carbonate, capable of releasing 1 g of phosphine.

Following oral ingestion, AlP reacts with water and stomach acid to produce phosphine gas, which may account in large part for its observed toxicity. Phosphine generated in the gastrointestinal tract is readily absorbed into the blood stream. Phosphine may denature or reduce oxyhemoglobin in addition to enzymes important for respiration and metabolism and may also affect cellular membranes. The exact mechanism of action of phosphine is still not clear. Some authors have claimed it to be an inhibitor of cytochrome oxidase in animals (Chefurka *et al*., 1976), while others showed reduction in catalase activity leading to free-radical toxicity in animals (Bolter *et al*., 1989) and humans (Chugh *et al*., 1996).

Symptoms of severe toxicity by AlP ingestion include diarrhea, cyanosis, breathing difficulty, pulmonary edema, respiratory failure, tachycardia, hypotension, dizziness, and death. The most common abnormality seen in the serum of 42% of poisoned patients was hypoxemia with severe metabolic acidosis (Khosla *et al*., 1992).

Phosphides, like phosphine, are toxic to the central nervous system, which is excited and then depressed. Arora *et al*. in 1995 reported that histopathological changes revealed varying degrees of congestion, similar to those produced by hypoxic injury. Phosphine was detected in tissues of the brain, liver, and kidneys.

The results of postmortem examinations of victims of phosphine poisoning generally indicate hypoxia with evidence of local trauma in the gastrointestinal tract, liver, kidneys, and central nervous system. The fatal dose of aluminium phosphide was stated to be in the range of 150–500 mg/70 kg by Chugh *et al*. in 1988. The mortality rate in clinical reports is stated to vary between 37–100% by different authors (Chopra *et al*., 1986; Kabra *et al*., 1988; Sepaha *et al*., 1985; Saraswat *et al*., 1985; Ram *et al*., 1985; Khosla *et al*., 1986; Mishra *et al*., 1989; Chugh *et al*., 1991, 1992, 1995).

The manner of death was reported to be suicidal in 87% of the cases by Dalbir *et al*. in 1985, 76% in a study by Chugh *et al*. and 100% in Jain *et al*. (2005) with all the cases being suicidal. Garry *et al*. reported in 1993 an accidental death related to phosphine exposure from stored grain fumigated with AlP pellets. The aluminium blood concentration was 713 pg/L, whereas normal laboratory values range from 2 to 42 pg/L. Ten control blood samples from various autopsies showed Al values between 9 and 15 μg/L using emission spectrometry, whereas blood in the deceased contained 100 times more. Similarly to the phosphine results, aluminium concentrations were found to be higher in the brain, adrenals, and liver. In urine and the kidneys, no difference from the usual published values was found, probably because death occurred shortly after ingestion.

Suicide was the most common mode of poisoning deaths, with overall mortality varying between 70–100%. Mortality is higher in those who consume more than two tablets and none of the patients survived who had ingested more than 3 tablets (Siwach *et al*., 1988; Singh *et al*., 1985; Raman *et al*. 1985). According to Karamjit *et al*. (2003) the pattern of poisoning varied in urban and rural areas, with a higher incidence of poisoning deaths in rural (64%) than in urban areas (36%). Aluminium phosphide was the most common poison consumed, being responsible for 50% of deaths, followed by insecticides 24% (Gupta *et al*., 2006). Poisoning deaths increased from 19% in 1996 to 24% in 2005. The age group most commonly affected was 16–25 years (49%). The male to female ratio was 1.9:1.0 and the rural to urban ratio was 1.5:1.0 (Gupta *et al*., 2006).

## Conclusion

Exposure to phosphine gas released from ALP fumigants increases risks of major morbidity and mortality. People handling this fumigant must be aware of its lethal aspects. An important preventive measure lies in better regulated supply of ALP, which otherwise is an excellent and safe fumigant as it leaves little residue on grain. Legislative and administrative measures have been suggested to restrict and modify its supply in India. Unfortunately there has been a failure in their implementation. Except the largest producer of it in India, it has been marketed as granulated powder in 10 g plastic sachets. Vendors and shop keepers should not sell the tablets to young people and children without proper verification and confirmation.
